# Peptide YY (PYY) Is Expressed in Human Skeletal Muscle Tissue and Expanding Human Muscle Progenitor Cells

**DOI:** 10.3389/fphys.2019.00188

**Published:** 2019-03-05

**Authors:** Brandon J. Gheller, Jamie E. Blum, Edward K. Merritt, Bethany P. Cummings, Anna E. Thalacker-Mercer

**Affiliations:** ^1^Division of Nutritional Sciences, Cornell University, Ithaca, NY, United States; ^2^Department of Kinesiology, Southwestern University, Georgetown, TX, United States; ^3^Department of Biomedical Sciences, Cornell University, Ithaca, NY, United States

**Keywords:** satellite cells, aging, stem cell, inflammation, metabolism, Peptide YY, muscle progenitor cells, skeletal muscle

## Abstract

Peptide YY (PYY) is considered a gut peptide with roles in post-prandial appetite and glucose regulation. Circulating PYY protein levels increase during aerobic exercise. Furthermore, people who have greater increases in muscle progenitor cells (*h*MPCs), the adult stem cell population responsible for skeletal muscle (SkM) repair, after resistance training have higher PYY transcript levels in SkM prior to training. Currently, examination of PYY expression patterns in SkM and/or *h*MPCs is lacking. Our objective was to identify the expression patterns of PYY in SkM and *h*MPCs. PYY and the associated Y receptors were analyzed in SkM biopsy tissue and cultured *h*MPCs from young and old human participants. Additional experiments to assess the role and regulation of PYY in *h*MPCs were performed. In SkM, PYY and one of the three Y receptors (Y1r) were detectable, but expression patterns were not affected by age. In expanding *h*MPCs, PYY and all three Y receptor (Y1r, Y2r, and Y5r) proteins were expressed in a temporal fashion with young *h*MPCs having greater levels of Y receptors at various time points. Exogenous PYY did not affect *h*MPC population expansion. *h*MPC PYY levels increased following the metabolic stimulus, 5-Aminoimidazole-4-carboxamide ribonucleotide (AICAR), but were not affected by the inflammatory stimulus, tumor necrosis factor alpha (TNFα). In conclusion, PYY and Y receptor expression are not impacted by age in SkM tissue but are reduced in old *vs.* young expanding *h*MPCs. Furthermore, endogenous PYY production is stimulated by low energy states and thus may be integral for skeletal muscle and *h*MPC responses to metabolic stimuli.

## Introduction

Peptide YY (PYY), a gut peptide released after food intake (De Silva et al., 2011), increases during aerobic exercise (Broom et al., 2008). The relationship between exercise and plasma PYY depends on exercise type and form of PYY measured (i.e., total PYY or PYY_3-36_) (Hazell et al., 2016). Post-exercise increases in PYY are posited to be from pancreatic or intestinal secretion via an undescribed mechanism (Hazell et al., 2016). There is evidence, however, skeletal muscle (SkM) may be a source of PYY. Basal PYY gene expression in SkM is greater in extreme hypertrophic responders to resistance exercise training compared to non-responders who displayed no/minimal hypertrophy (Thalacker-Mercer et al., 2013). The extreme responders have more muscle progenitor cells (MPCs), the adult stem cell population responsible for SkM repair (Petrella et al., 2008). In humans, PYY acts through the Y family of Gα_i_-protein coupled receptors (Y1r, Y2r, and Y5r). PYY exists in two forms, PYY_1-36_ has affinity for all three receptors while PYY_3-36_, the cleaved form of PYY_1-36,_ has increased affinity for Y2r (Berglund et al., 2003). Expression of the Y receptors is documented in rodent myoblasts (Zhang et al., 2010) but, to our knowledge, their expression in human MPCs (*h*MPCs) and SkM has not been identified.

In this report, we provide the first thorough examination of PYY and Y receptor expression in human SkM and *h*MPCs. Additionally, we elucidate temporal and age-related differences in the expression of PYY and the Y receptors throughout *h*MPC population expansion. Finally, we identify that PYY levels in *h*MPCs are altered by compounds that affect *h*MPC metabolism and consequently population expansion such as the activator, 5-Aminoimidazole-4-carboxamide ribonucleotide (AICAR).

## Materials and Methods

### Participants

Young (21–40 years) and old adults (65–80 years) were recruited from the Ithaca, New York and Boone, North Carolina areas (participant characteristics in Supplementary Table S1) in a manner previously published (Riddle et al., 2018a,b). The Cornell University and Appalachian State University Institutional Review Boards approved the protocols. All subjects gave written informed consent in accordance with the Declaration of Helsinki.

#### Skeletal Muscle Biopsies and Primary *h*MPC Culture

Skeletal muscle tissue and primary *h*MPCs were obtained using previously published methods (Riddle et al., 2018b). Briefly, SkM tissue was taken from the vastus lateralis using the percutaneous biopsy technique. Tissue was snap-frozen in liquid nitrogen and stored at -80°C (SkM) or stored in Hibernate^®^-A medium (Invitrogen) at 4°C until tissue disassociation. *h*MPCs were isolated as previously described (Xu et al., 2015; Riddle et al., 2018b) and were verified to be positive for the MPC specific transcription factor MyoD (Supplementary Figure S1). Passage six *h*MPCs were cultured in a 5% CO_2_ atmosphere at 37°C on collagen (Type I, Rat Tail, Corning) coated plates. Only female *h*MPCs were used due to sample availability.

### SkM RNA and Protein Extraction

RNA was extracted from SkM tissue using Trizol Reagent (Ambion). SkM tissue was homogenized in RIPA buffer containing protease and phosphatase inhibitors (cOmplete and PhosStop, Roche) to isolate protein.

### Radioimmunoassay

Total human PYY in SkM protein lysate was measured using a commercially available radioimmunoassay (EMD Millipore, PYYT-66HK). PYY values were normalized to the wet weight (mg) of tissue.

### Quantitative RT-PCR

Gene expression for PYY and the Y receptors was measured using quantitative RT-PCR. The Taqman Gene Expression System (Applied Biosystems) was used to measure mRNA expression levels of: PYY (HS00358823), Y1r (Hs00268565), Y2r (Hs01921296), Y5r (Hs01883189), and IL-6 (Hs00985639). All samples were normalized to 18S (Hs99999901) expression. Data were expressed as delta C_p_ values.

### Immunoblotting

Y receptor protein levels in SkM were determined by immunoblotting. Protein lysates were subjected to deglycosylation under denaturing reaction conditions according to manufacturer’s instructions (P0704, New England Biolabs). Following deglycosylation, lysates were heated at 60°C and cooled to room temperature. 20 μg of protein was loaded in 10% SDS gels and transferred to PVDF membranes using Stain-Free technology (Bio-Rad). Membranes were incubated in primary antibodies Y1r, Y2r, and Y5r (NBP1-59008, NB100-56480, NB100-1538, respectively, Novus Biologicals) diluted (1:1000) in a chemiluminescent blocking buffer (blØk^TM^ – CH, Millipore) overnight at 4°C. After the overnight incubation, membranes were washed with TBST before incubation with appropriate secondary antibody (anti-rabbit, Proteintech or anti-goat, Thermo Scientific) at a 1:25000 dilution in blocking buffer at room temperature. Membranes were visualized after incubation in SuperSignal^TM^ West Femto (Thermo Scientific) on the Bio-Rad ChemiDoc MP.

### *h*MPC Treatments

For *h*MPC population expansion experiments, recombinant human PYY_1-36_, PYY_3-36_ (Phoenix Pharmaceuticals), or vehicle control (sterile H_2_O) was administered every 24. For TNFα (Millipore Sigma) and AICAR (Cell Signaling Technology) experiments, *h*MPCs were treated with the compound or vehicle control (sterile H_2_O or DMSO) for the specified time, then fixed with 4% formaldehyde for In-Cell Western analysis.

The number of live cells was measured every 24 h by co-staining cells with Hoechst 33342 (to identify number of nuclei) and Propidium Iodide (to identify dead cells).

### In-Cell Western Assay

Fixed *h*MPCs were permeabilized with 0.1% Triton in PBS. Cells were incubated in primary antibodies, specific for PYY (ab22663, Abcam, Supplementary Figure S2) and Y1r, Y2r, and Y5r diluted (PYY, 1:1000; Y receptors, 1:100) in Odyssey Blocking Buffer (LI-COR), overnight at 4°C. Cells were washed with 0.1% PBST and incubated at room temperature in secondary antibody (IRDye^®^ 800CW anti-rabbit, LI-COR or IRDye^®^ 800CW anti-goat, LI-COR) diluted 1:400 in Odyssey Blocking Buffer. Fluorescence intensity, representing protein levels, was measured using the Odyssey Imaging System (LI-COR). Protein levels were normalized to number of cells via Hoechst 33342 staining and counting using the Celigo^®^ Imaging Cytometer (Nexcelom Bioscience).

### Immunocytochemistry

Fixed *h*MPCs were permeabilized with 0.1% Triton in PBS and incubated in primary embryonic myosin heavy chain (eMHC) antibody (DSHB Hybridoma Product F1.652, F1.652 was deposited to the DSHB by Blau, Helen M.) (Webster et al., 1988) and either primary Y2r or Y5r antibodies diluted (1:100) in eMHC supernatant, overnight at 4°C. Cells were washed with PBS and incubated in secondary antibody (AlexFluor^®^ 568 anti-mouse and AlexFluor^®^ 488 anti-rabbit, Life Technologies) at 37°C, diluted 1:1000 in 1% goat serum. Nuclei were counterstained with Hoechst 33342.

### Statistics

All statistical analyses were performed in GraphPad 6.0. Protein and gene expression between young and old, were compared using an unpaired *t*-test. A two-way analysis of variance (ANOVA), with main factors of age and time, was used to discern differences in protein levels in *h*MPCs. To compare live cell count after treatment a two-way, repeated measures ANOVA with treatment and time being the main factors was conducted. To evaluate the effect of TNFα and AICAR on *h*MPC PYY levels, either a two-way ANOVA (treatment and age) or a one-way repeated measures ANOVA (treatment) was performed. When the interaction term was significant, a Tukey HSD *post hoc* test was performed.

## Results

### PYY and Y Receptor Expression in Human SkM and *h*MPCs

**FIGURE 1:**
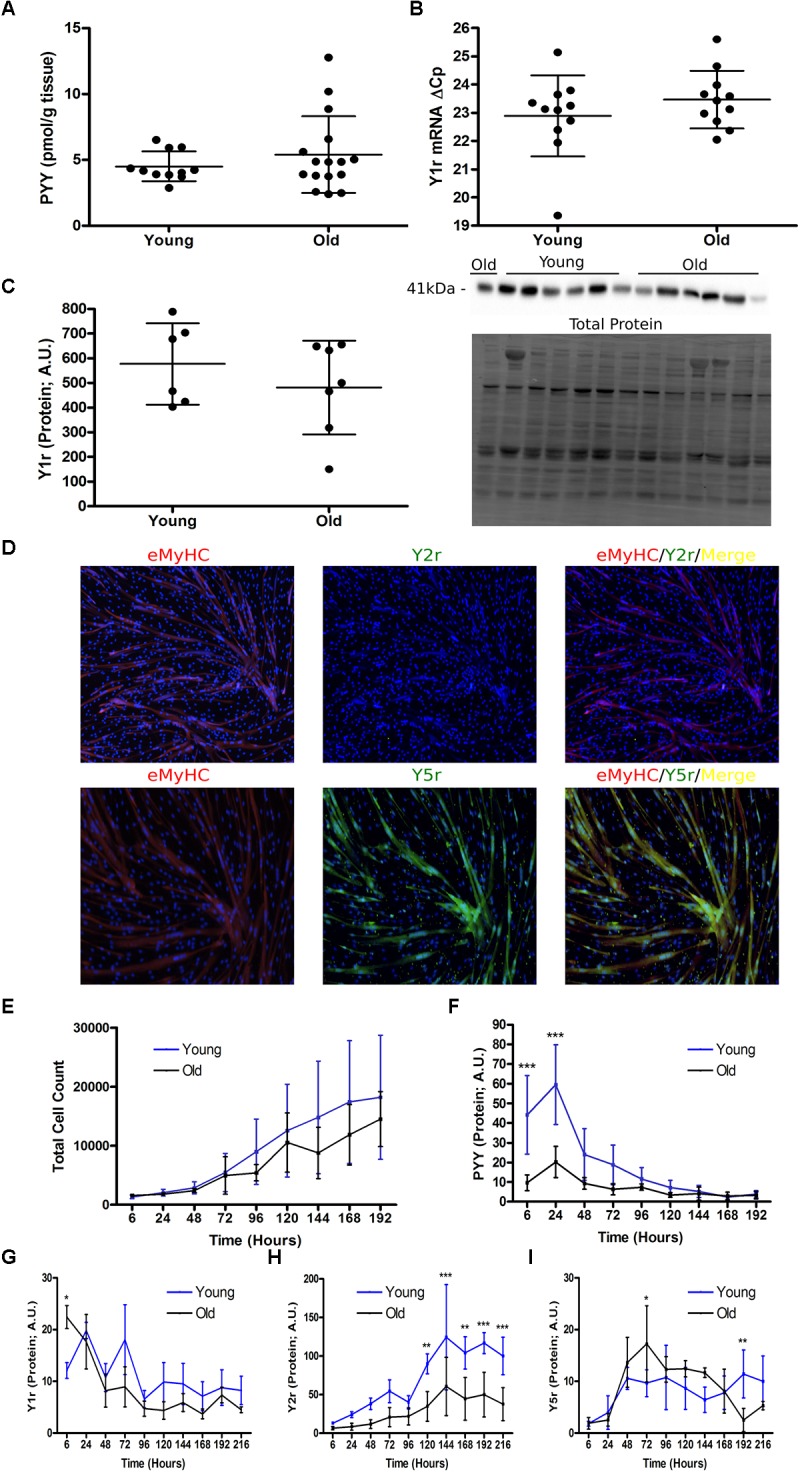
Characterization of PYY and the Y receptors in human skeletal muscle and expanding human muscle progenitor cells. Expression of **(A)** PYY protein expression in SkM of young (*n* = 11) and old (*n* = 16) individuals. SkM Y1r expression at the **(B)** gene (*n* = 27) and **(C)** protein level (*n* = 11). **(D)** Representative microscopy images of *h*MPCs co-stained with a DNA stain (Hoechst 33342, blue), Y2r or Y5r (green), and embryonic myosin (red). **(E)** Expansion curves of cultured *h*MPCs isolated from young (*n* = 5) and old (*n* = 5) donors. Protein expression of **(F)** PYY, **(G)** Y1r, **(H)** Y2r, and **(I)** Y5r in expanding young (*n* = 5) and old (*n* = 5) *h*MPC cultures. **A–C**: unpaired *t*-test. **E–I**: Two-way ANOVA, main effect of age, time, age × time interaction (stars denote results of Tukey’s HSD *post hoc* test performed when interaction term was significant) ^∗^*p* < 0.05, ^∗∗^*p* < 0.01, and ^∗∗∗^*p* < 0.001. Data are expressed as mean ± SEM. SkM, skeletal muscle; PYY, Peptide YY; Yr, Y receptor; *h*MPC, human muscle progenitor cell.

Peptide YY protein and Y1r mRNA and protein, but not Y2r and Y5r (data not shown), were detectable in SkM, but were not different between young and old individuals (Figure 1A–C). To validate that Y2r and Y5r are not expressed in SkM, we differentiated *h*MPCs for 7 days, until eMHC was expressed, and used immunocytochemistry to visualize Y2r and Y5r expression (Figure 1D). Y2r could not be visualized in myotubes, however, Y5r was detectable and colocalized with eMHC.

In young (*n* = 5) and old (*n* = 5) *h*MPCs, PYY was detectable and declined throughout 192 h of population expansion (*p* < 0.05, Figure 1F). PYY protein levels were greater in *h*MPCs from young (vs. old) at 6 h and 24 h after seeding (*p < 0.*05, Figure 1F). Y1r protein levels declined throughout population expansion (*p* < 0.05, Figure 1G). Young *h*MPCs had greater levels of Y1r protein than old *h*MPCs at 6 h after seeding (*p* < 0.05, Figure 1G). Y2r protein levels increased with population expansion (Figure 1H). Young (vs. old) *h*MPCs had higher levels of Y2r from 120 to 216 h after seeding (*p* < 0.01, Figure 1H). Compared to old *h*MPCs, young *h*MPCs expressed greater levels of Y5r protein at 72 h (*p* < 0.05) and 192 h after seeding (*p* < 0.01, Figure 1I). Collectively these data demonstrate that SkM tissue PYY levels do not differ between young and old; however, PYY and the Y receptors are higher in young compared with old *h*MPCs. This age difference between SkM and cultured *h*MPCs may be related to differences only observed during dynamic conditions (i.e., population expansion).

### Role and Regulation of PYY During *h*MPC Population Expansion

**FIGURE 2:**
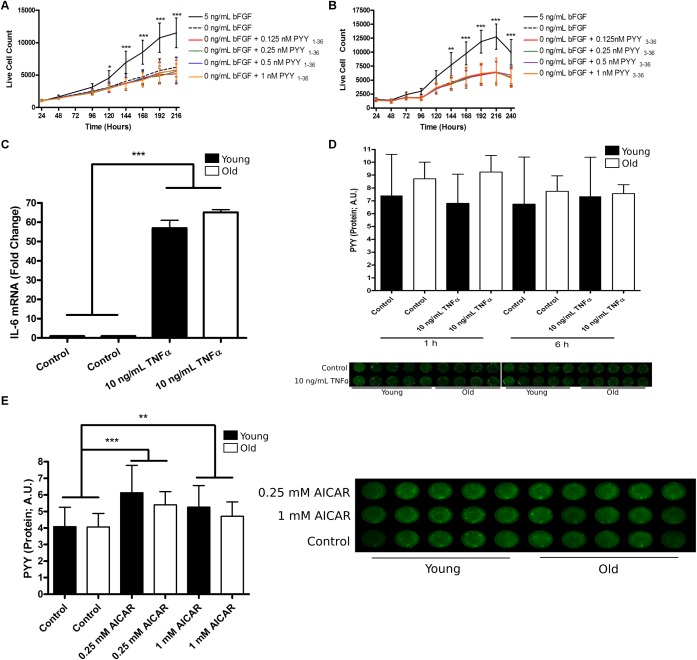
The role and regulation of PYY in human muscle progenitor cells. **(A**) PYY_1-36_ or **(B)** PYY_3-36_ do not affect *h*MPC expansion. **(C)** The effect of 6 h of 10 ng/mL TNFα treatment on IL-6 mRNA levels in young (*n* = 5, black bars) and old (*n* = 5, open bars) *h*MPCs. **(D)** PYY protein levels in young (*n* = 5) and old (*n* = 5) *h*MPCs after 1 and 6 h of 10 ng/mL TNFα exposure. **(E)** PYY protein levels in young (*n* = 5) and old (*n* = 5) *h*MPCs after 6 h of 0.25 or 1 mM AICAR treatment. **A,B**: Two-way ANOVA, main-effect of treatment, time, and treatment-by-time interaction and **C–E**: Two-way ANOVA, main-effect of treatment, age, and treatment-by-age interaction (asterisks denote results of Tukey’s HSD *post hoc* test performed when interaction term was significant). ^∗^*p* < 0.05, ^∗∗^*p* < 0.01, and ^∗∗∗^*p* < 0.001. Data are expressed as mean ± SEM. PYY, Peptide YY; Yr, Y receptor; hMPC, human muscle progenitor cell; TNFα, tumor necrosis factor alpha; AICAR, 5-Aminoimidazole-4-carboxamide ribonucleotide.

Noting the transient expression of PYY and the Y receptors during *h*MPC population expansion, we hypothesized that exogenous PYY promotes *h*MPC population expansion. Basic fibroblast growth factor (bFGF) and the PYY-related peptide, neuropeptide Y, have been shown to have similar, but not necessarily additive, effects on human embryonic cell proliferation (Son et al., 2011); therefore, for some of these experiments, bFGF was removed. To determine the effect of PYY on *h*MPC population expansion, *h*MPCs from young donors (*n* = 5) were cultured with or without bFGF and supplemented with varying doses of PYY_1-36_ or PYY_3-36_. *h*MPCs grown in the presence of PYY_1-36_ (Figure 2A) or PYY_3-36_ (Figure 2B) and without bFGF showed no increase in population expansion compared to a no bFGF control. Therefore, exogenous PYY does not appear to regulate *h*MPC population expansion.

Alternatively, we hypothesized that because PYY and Y receptor levels were highest during most rapid population expansion (Figure 1E–I), endogenous PYY levels may be regulated by factors that affect this process. First, we considered TNFα, an inflammatory cytokine that promotes *h*MPC population expansion (Miller et al., 1988). After *h*MPCs from young (*n* = 4) and old donors (*n* = 5) entered exponential growth (96 h after seeding) they were treated with 10 ng/mL of TNFα. This treatment increased *IL-6* mRNA levels by ∼50-fold (Figure 2C) within 6 h. TNFα exposure for 1 h (*p* = 0.88) or 6 h (*p* = 0.66) had no effect on *h*MPC PYY levels regardless of age (Figure 2D).

Next we treated *h*MPCs with AICAR, an activator of AMPKα; AMPKα activity has been implicated in the regulation of MPC population expansion (Pavlidou et al., 2017). *h*MPCs were treated with either 0.25 or 1 mM AICAR for 6 h after cells entered exponential growth (96 h after seeding). 6 h of 0.25 mM AICAR increased *h*MPC PYY levels by 42% (*p* = 0.0001) and 1 mM produced a 30% increase (*p* = 0.02, Figure 2E). We observed no age-related differences.

## Discussion

This report presents the first examination of PYY and Y receptor expression in human skeletal muscle, using two systems: SkM and *h*MPCs. Under resting conditions PYY and Y1r, but not Y2r or Y5r, are expressed in SkM. Our data suggest that *h*MPC PYY expression is regulated by metabolic state. Circulating PYY concentrations increase after exercise to a modest degree (Hazell et al., 2016), but the source of this PYY is unknown. Our results support that SkM-derived PYY could be one contributor. The lack of difference between PYY and Y receptor levels in young and old SkM suggests no age-related difference in the activity of this peptide during steady state conditions, however, we detected age-related differences in expanding *h*MPCs. Therefore, it is likely age-related differences exist during dynamic conditions such as during regeneration. Y1r, Y2r, and Y5r were expressed during *h*MPC expansion, only Y1r and Y5r were detectable in either SkM tissue or differentiated *h*MPCs, suggesting PYY signaling depends on state of differentiation.

*h*MPC PYY levels were shown to be unaffected by a pro-expansion stimulus (TNFα) but were increased by an anti-expansion stimulus (AICAR). The increase in *h*MPC PYY levels could be due to intracellular PYY synthesis and storage. Alternatively, increased *h*MPC PYY levels could be due to increased binding of PYY from the media to the Y receptors on the cell surface. This explanation is unlikely as bovine PYY differs from human PYY (71% homology) and differences were seen between young and old *h*MPCs under similar culture conditions. Alternatively, as PYY is traditionally considered a secretory peptide (Psichas et al., 2015) it could be posited that AICAR treatment resulted in the synthesis, secretion, and binding of PYY in an autocrine manner.

## Conclusion

In conclusion, we present the most comprehensive evidence to date that PYY is produced by post-mitotic SkM tissue and expanding *h*MPCs. Furthermore, we identify AICAR treatment, which negatively affects *h*MPC population expansion, as an inducer of PYY expression in *h*MPCs. We propose that *h*MPC endogenous PYY and Y receptor activity may be integral for skeletal muscle and *h*MPC responses to metabolic stimuli.

## Data Availability

All datasets generated for this study are included in the manuscript and/or the Supplementary Files.

## Author Contributions

BG and AT-M designed the experiments. BG, JB, EM, BC, and AT-M collected, analyzed, and interpreted the data.

## Conflict of Interest Statement

The authors declare that the research was conducted in the absence of any commercial or financial relationships that could be construed as a potential conflict of interest.

## Supplementary Material

FIGURE S1 Passage 6 hMPCs are MyoD positive. Representative microscopy images of passage 6 *h*MPCs co-stained with a DNA stain (Hoechst 33342, blue) and the MPC-specific transcription factor MyoD (red).

FIGURE S2 Validation of the PYY antibody for the In-Cell Western assay. In-Cell Western images demonstrating the specificity of the PYY antibody used for all experiments to recombinant PYY and not the closely related NPY.

TABLE S1 Baseline participant characteristics.
