# Supplementary figures and images for: Peptide YY (PYY) Is Expressed in Human Skeletal Muscle Tissue and Expanding Human Muscle Progenitor Cells
Source: Front Physiol. 2019 Mar 5;10:188. doi: 10.3389/fphys.2019.00188 (PMC6412030; doi:10.3389/fphys.2019.00188)

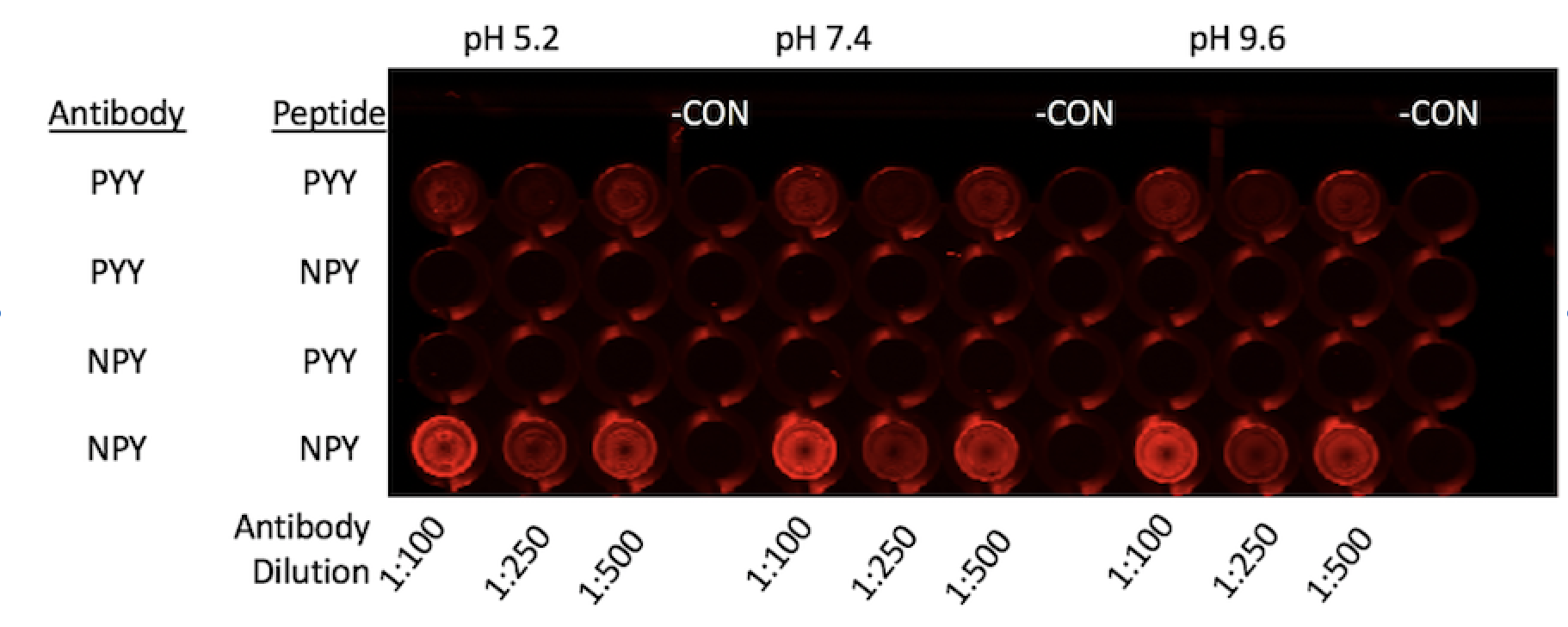

Supplement: FIGURE S2 — Validation of the PYY antibody for the In-Cell Western assay. In-Cell Western images demonstrating the specificity of the PYY antibody used for all experiments to recombinant PYY and not the closely related NPY. [file Image_2.TIFF]
