# Supplementary material for: Peptide YY (PYY) Is Expressed in Human Skeletal Muscle Tissue and Expanding Human Muscle Progenitor Cells
Source: Front Physiol. 2019 Mar 5;10:188. doi: 10.3389/fphys.2019.00188 (PMC6412030; doi:10.3389/fphys.2019.00188)
Supplement: TABLE S1 — Baseline participant characteristics. [file Table_1.DOCX]

**Supplementary Table 1.** *Baseline Participant Characteristics*

|  | **Figure 1A-B** | | **Figure 1C** | | **Figure 1E-I** | | **Figure 2A-B** | | **Figure 2C-E** | |
| --- | --- | --- | --- | --- | --- | --- | --- | --- | --- | --- |
|  | Young (n=11) | Old (n=16) | Young (n=6) | Old (n=5) | Young (n=5) | Old (n=5) | Young (n=5) | Old (n=0) | Young (n=5) | Old (n=5) |
| **Sex (M/F)** | 6/5 | 4/12 | 0/6 | 0/5 | 0/5 | 0/5 | 0/5 | - | 0/5 | 0/5 |
| **Age (y)** | 27.1 ± 5.0 | 70.3 ± 5.8 | 30.7 ± 4.0 | 70.6 ± 20.4 | 28.2 ± 4.0 | 70.4 ± 7.6 | 29.4 ± 3.6 | - | 29.4 ± 3.6 | 70.4 ± 7.6 |
| **Height (m)** | 1.7 ± 0.1 | 1.7 ± 0.1 | 1.7 ± 0.0 | 1.6 ± 0.1 | 1.7 ± 0.0 | 1.6 ± 0.1 | 1.7 ± 0.0 | - | 1.7 ± 0.0 | 1.6 ± 0.1 |
| **Weight (kg)** | 70.4 ± 16.3 | 70.1 ± 9.7 | 70.2 ± 14.1 | 63.4 ± 12.9 | 64.4 ± 13.5 | 63.6 ± 9.7 | 67.3 ± 16.9 | - | 67.3 ± 16.9 | 63.6 ± 9.7 |
| **BMI** | 23.4 ± 3.8 | 25.6 ± 3.5 | 25.4 ± 4.7 | 24.0 ± 3.8 | 23.3 ± 4.0 | 25.0 ± 4.1 | 24.5 ± 5.7 | - | 24.5 ± 5.7 | 25.0 ± 4.1 |

All data are presented as means ± SD.
